# Targeted Inter-Homologs Recombination in Arabidopsis Euchromatin and Heterochromatin

**DOI:** 10.3390/ijms222212096

**Published:** 2021-11-09

**Authors:** Shdema Filler-Hayut, Kiril Kniazev, Cathy Melamed-Bessudo, Avraham A. Levy

**Affiliations:** Department of Plant and Environmental Sciences, Weizmann Institute of Science, Rehovot 7610001, Israel; shdema@mit.edu (S.F.-H.); kiril.knz@gmail.com (K.K.)

**Keywords:** homologous recombination, DNA repair, CRISPR Cas9, chromatin

## Abstract

Homologous recombination (HR) typically occurs during meiosis between homologs, at a few unplanned locations along the chromosomes. In this study, we tested whether targeted recombination between homologous chromosomes can be achieved via Clustered Regulatory Interspaced Short Palindromic Repeat associated protein Cas9 (CRISPR-Cas9)-induced DNA double-strand break (DSB) repair in *Arabidopsis thaliana*. Our experimental system includes targets for DSB induction in euchromatic and heterochromatic genomic regions of hybrid F1 plants, in one or both parental chromosomes, using phenotypic and molecular markers to measure Non-Homologous End Joining and HR repair. We present a series of evidence showing that targeted DSBs can be repaired via HR using a homologous chromosome as the template in various chromatin contexts including in pericentric regions. Targeted crossover was rare, but gene conversion events were the most frequent outcome of HR and were found in both “hot and cold” regions. The length of the conversion tracts was variable, ranging from 5 to 7505 bp. In addition, a typical feature of these tracks was that they often were interrupted. Our findings pave the way for the use of targeted gene-conversion for precise breeding.

## 1. Introduction

DNA Double Strand Breaks (DSBs) may occur in somatic plant cells under exposure to ultraviolet light, ionizing radiation, chemical mutagens, through activation of endonucleases, or during replication when replication fork collapse. Unrepaired breaks may have deleterious effects such as chromosome loss, gamete sterility, or even cell death. Thus, it is not surprising that the complex networks of genes responsible for sensing and repairing these breaks are conserved through evolution from yeast to plants and vertebrates. DSB repair mechanisms can be divided into *cis*-repair mechanisms, which involve ligation of the two broken DNA ends without any homologous template for repair, and *trans*-repair mechanisms, where the broken DNA repair is based on homologous sequence such as sister chromatid, homologous chromosome, or any other exogenous or endogenous homologous sequence.

The *cis*-repair mechanisms include classical NHEJ (cNHEJ), alternative NHEJ (a-NHEJ), and Single Strand Annealing (SSA). The NHEJ mechanisms are producing an accurate DNA repair product, identical to the DNA before DSB, or a product with small insertion/deletion (indels) at the break site (see [1,2] for review). *Trans*-repair mechanisms (Synthesis Dependent Strand Annealing [SDSA] and double Holliday Junction pathway) are based on homologous recombination (HR). The end-product of the SDSA process is a non-crossover (also called gene conversion). The double Holliday Junction (dHJ) pathway can lead to two optional repair products. The dHJ intermediate may be resolved by nicking of the two strands at each junction to create non-reciprocal (non-crossover) or reciprocal (crossover) products, depending on nicking orientation. The exact mechanism of dHJ resolution is still under debate (see [3,4,5,6] for review).

In plants, it is generally thought that most somatic DSBs are repaired by the NHEJ mechanisms [7]. A study testing the kinetics of NHEJ repair in Arabidopsis, showed that in root tip tissue, after ionizing irradiation, c-NHEJ acts very rapidly and prevents the activation of another repair mechanism (a-NHEJ, SSA or HR-based) [8]. In agreement, NHEJ repair products were found to be the dominant repair products in somatic plant tissues that underwent DNA DSBs induction by site-specific meganucleases [9], transposon excision [10], and custom-designed nucleases, such as zinc-finger nucleases (ZFNs) [11], transcription activator-like effector nucleases (TALENs) [12], and Clustered Regulatory Interspaced Short Palindromic Repeat associated protein Cas9 (CRISPR-Cas9) [13,14,15,16].

Repair based on homologous recombination depends on the availability of a DNA molecule with homology to the sequence flanking the DSB site. In the presence of an exogenous DNA donor (template for HR based repair), targeted induction of DSB using meganucleases [17], ZFNs [18], TALEN [19], or CRISPR-Cas9 [20] can lead to somatic recombination, indicating that the HR repair machinery is active in plant somatic cells. Studies in both Arabidopsis [21] and barley [22] showed that the sister chromatid can also serve as a template for HR repair in somatic cells. In a previous study, we have demonstrated that induction of DSBs using CRISPR-Cas9 can lead to up to 14% of inter-homolog recombination (IHR) in somatic cells at the *Psy1 locus* of tomato [23]. In Arabidopsis, a recent study also showed that IHR can be used for DSB repair, generating gene conversion, or a gene drive [24]. This was shown in a transgenic system and IHR-mediated DSB repair has not been analyzed yet for endogenous loci in Arabisopsis.

Classic breeding procedures are based on natural meiotic crossover events. The distribution of these events was documented by high-resolution maps in a few plant species including Arabidopsis [25,26], maize [27], and wheat [28,29]. In general, crossover frequency was found to be higher in sub-telomeric regions (maize and wheat) or whole chromosome arm (Arabidopsis) and reduced in pericentric regions or at the telomeres (wheat, maize, and Arabidopsis). In plants, crossovers were found to be associated with gene promoters, chromatin features characterizing open chromatin regions, such as low DNA methylation, low nucleosome occupancy, and chromatin modifications such as H2A.Z, H3K4me3, and with three sequence motifs: A-rich sequences, CTT motifs, and CCN motifs [25,26,30,31]. Meiotic crossover is initiating with the induction of hundreds of DNA DSBs by the SPO11protein [32]. The distribution of these breaks was documented in yeast [33], mice [34], maize [27], and Arabidopsis [31], and is found to be non-uniform along the chromosome as well as to correlate with crossover sites.

Here, we report on the repair products of DSBs induced by CRISPR-Cas9 in several targets located in euchromatic and heterochromatic regions of the Arabidopsis genome. IHR events were found in both euchromatin and heterochromatin. Gene conversion was the main outcome of DSB-induced IHR while crossovers were rare. Gene conversion products of DSB-induced IHR were germinally transmitted in all chromatin contexts mentioned above, with conversion tract size ranging from a few base-pairs to few kilo-base-pairs, with both simple and complex conversion tracts. In addition, we characterized an event of targeted crossover (CO) that was germinally transmitted.

## 2. Results

### 2.1. Selection of gRNA for DSB-Induction

**Figure 1:**
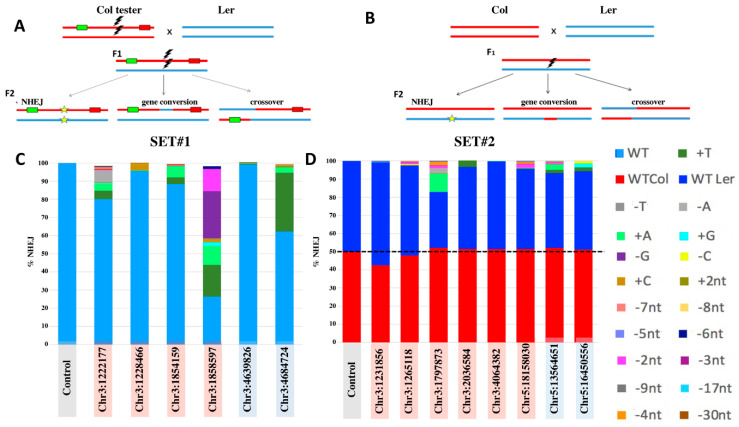
Schematic description of crosses, DSB induction and NHEJ frequency in different targets. (**A**) Experimental scheme for the analysis of somatically induced DNA DSB repair. The Col parent is heterozygote for the meiotic tester Col3-4/20. It is transformed with a construct containing Cas9 and a gRNA that targets a unique site in between the RFP and GFP markers (shown as red and green rectangles, respectively). It was crossed with Landsberg WT plants. In the resulting F1 DSB (lightning) induction can cleave both Columbia and Landsberg chromosomes at allelic targets. Each construct includes a single gRNA belonging to Set#1 (Supplementary Table S1). DNA DSBs can be repaired via NHEJ (recognized as indels indicated by a star), gene conversion, or crossover. The homologous chromosomal segments are shown as a red line for Columbia and as a blue line for Landsberg. (**B**) Plants of Columbia transformed with both Cas9 and a specific gRNA belonging to Set#2 (Supplementary Table S1) were crossed with Landsberg WT plants. In the resulting F1 plants, DSB (lightning) induction is allele specific to Landsberg allele. (**C**,**D**) show the % of NHEJ for guides of Set#1 and Set#2, respectively. DNA was amplified around the DSB target site and the resulting PCR fragments were sequenced by Illumina platform and the resulting reads analyzed with Cas-analyzer [37]. The NHEJ % represents the percent of reads containing indels out of the total number of reads. Each bar represents an average of the indel frequency of a pool of ~100 F2 seedlings (10 seedlings from 10 plants). In the x-axis each target is shown by its chromosome and coordinates. The red and blue boxes indicate hot (euchromatin) and cold (heterochromatin) targets respectively. The legend at the right describes the color and name of the most prominent footprints.

In order to test and characterize the repair of DSBs by IHR, we selected 26 targets in the Arabidopsis genome and worked in a Columbia (Col) X Landsberg (Ler) genetic background that provided the polymorphism needed to analyze recombination events. gRNAs were designed to both euchromatic and heterochromatic regions on the basis of features typical of hot or cold recombination spots, such as nucleosome occupancy, CG methylation [35], and H3K4me3, as well as sequence motifs [36] that are associated with meiotic recombination [25] (Supplementary Table S1). DSBs were induced in somatic tissues of F1 plants by co-expression of gRNAs with Cas9, and F2 seeds were collected for further molecular analyses of germinally transmitted genetic changes following DSB repair (See Figure 1). Two schemes of crosses were conducted to study IHR-mediated DSB repair: one scheme (Figure 1A) focused on a specific region of chromosome 3 where we had a pair of linked markers (see details below), while in the other cross (Figure 1B) we used gRNAs throughout the genome including in pericentric regions.

We tested two sets of targets (Supplementary Table S1). Set#1 consisted of 12 gRNAs located in the region between two markers at a distance of 5 Mbp (GFP and RFP seed-fluorescence markers) in the distal part of chromosome3 long arm, in the meiotic tester Col3-4/20 that we previously developed [38]. The meiotic tester enabled us to select CO events that were germinally transmitted in F2 seeds (red [RFP] only or green [GFP] only seeds) and to test if crossover occurred at the expected break site. Four loci had features of cold spots, corresponding to heterochromatin embedded within euchromatin, with a high degree of cytosine methylation and high nucleosome occupancy (Supplementary Table S1) and eight loci had a euchromatic epigenetic context. The gRNAs could cleave both Columbia (Col) and Landsberg (Ler) targets (Supplementary Table S1).

Set#2 consisted of 14 gRNAs targeting various chromosomal regions, including in heterochromatin from pericentric regions, and were designed to be chromosome-specific, cleaving only the Ler chromosome. Taking advantage of SNPs between the Landsberg and the Columbia ecotypes, targets were chosen with an optimized PAM sequence on the Landsberg background (NGG) and an altered PAM sequence on the Columbia background (NGH) which prevents Cas9 cleavage [39] (Figure 1B and Supplementary Table S1).

Fourteen out of the 26 gRNAs showed evidence for DSB induction as seen by NGS data on NHEJ indels at the break site (Figure 1C,D). The remaining 12 gRNAs, listed in Supplementary Table S1, did not show any sign of NHEJ footprints at the target site. In Set#1, four targets with NHEJ footprints were from euchromatin with features of hot regions and two targets were from regions with features of heterochromatin embedded in euchromatin (Figure 1C, Supplementary Table S1). Editing percentage ranged between 0.8 and 73.6%. In Set#2, chromosome specificity was confirmed by the lack of indel footprints in the Col chromosome (Figure 1D). Six out of the seven euchromatin targets, and two out of the seven heterochromatic targets showed NHEJ repair activity at the targeted site (Figure 1D, Supplementary Table S1). Editing percentage ranged between 0.4 and 38.5%. Both euchromatic and heterochromatin DSBs were mainly repaired by small insertions or deletions of 1–2 nucleotides.

### 2.2. Analysis of Targeted IHR Events

**Figure 2:**
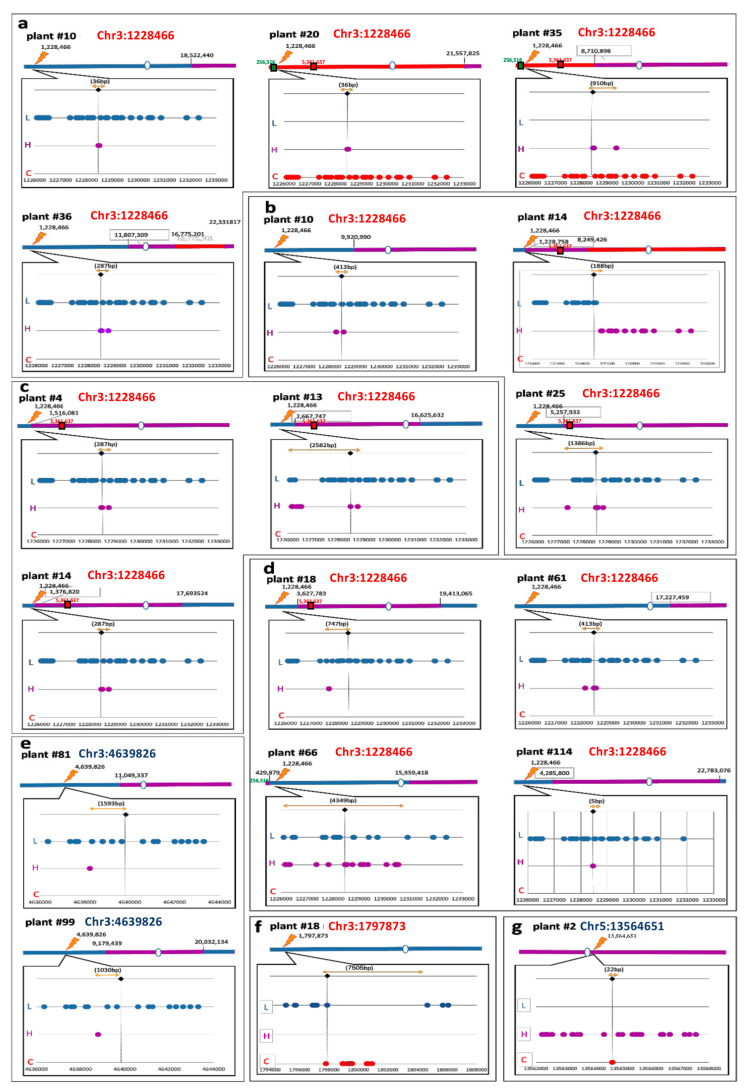
Targeted IHR Events. Maps of recombinant F2 plants. The chromosome# and coordinates of each target are indicated in red and blue for each plant analyzed in euchromatic and heterochromatic region, respectively. The genotyping of the DSB region was performed by whole genome sequencing. For each plant, a continuous line represents the entire chromosome, homozygous chromosomal sequences are indicated by a red line for Columbia and by a blue line for Landsberg. Heterozygous regions are indicated by a purple line, and the centromeres by a hollow circle. The DNA DSB location is indicated by lightning and the coordinates of crossovers along the chromosome are indicated above each crossover site. The “bubble” below each chromosome, represents the magnification of the induced DNA DSB region. Each SNP in the graph is represented by a red/purple/blue dot corresponding to Columbia homozygous/heterozygous/Landsberg homozygous reads, respectively. The DSB site is indicated by black diamond and vertical black dashed line. The distance between heterozygous SNPs, enables assessing the minimal length of the conversion tract. The tract is represented by a yellow arrow above the DSB site, and its length indicated in the brackets. The GFP and RFP position from the Columbia tester are shown as green and red squares, respectively, with their coordinates above them (256,516 bp for GFP and 5,361,637 for RFP). Results are shown for individual F2 plants, progeny of F1 plants for (**a**) target Chr3:1228466 progeny of F1plant#1, (**b**) target Chr3:1228466 progeny of F1 plant#2-RFP only seeds, (**c**) target Chr3:1228466 progeny of F1 plant#5-RFP only seeds, (**d**) target Chr3:1228466 progeny of F1 plant#7, (**e**) target Chr3:4639826 progeny of F1 plant#4, (**f**) target Chr3:1797873 progeny of F1 plant #4b, and (**g**) target Chr5:13564651 progeny of F1 plant #1d.

HR-repair products were analyzed in the F2 progeny of the F1 hybrid plants (Figure 1A,B). Regarding targets of Set#1 (Figure 1C), F2 plants with RFP only or GFP only seeds were selected (Supplementary Table S2), as expected for crossover events between the two markers in the F2 seeds. In addition, non-sorted plant populations were also grown to allow a non-biased characterization of DSB repair transmitted to the next generation (Supplementary Table S2). Whole genome sequencing was performed to enable the analysis of the whole region around the targeted DSBs. All F2 plants were grown without antibiotic selection and DNA was extracted from rosette leaves. DNA from each plant was sonicated to ~300 bp fragments, barcoded with a unique barcode that allows downstream sorting of the reads to their original sample (1 barcode per plant) and sequenced by Illumina paired-end sequencing. For each F2 plant, reads were aligned to the Arabidopsis genome, crossover events were detected using hidden Markov custom script and confirmed using the IGV browser [40] for gene conversion detection. We chose 13 different F1 plants representing three targets (Chr3:1228466, Chr3:1854159 and Chr3:4639826). In total we sequenced the whole genome of 236 F2 plants. The presence of NHEJ, non-crossover, and crossover signatures at the induced DSB site was characterized for each F2 plant (Supplementary Table S2). In one, out of 17 F2 plants analyzed for target Chr3:1228466_RFP_only, we identified a crossover event located at the induced DSB (Figure 2b, plant# 14, Supplementary Table S2).

To confirm the exact crossover site, we grew the F3 offspring of this plant, selected a homozygous plant in the crossover region and sequenced a 5 Kb fragment flanking the DSB site using PacBio^®^ (Supplementary Figure S1). The distance between the induced DNA DSB site and the crossover site was 188 bp (crossover site was estimated by averaging the coordinates of the indicative SNPs that flank the CO region and the coordinates of the DSB as indicated in Supplementary Table S1). This distance can be explained by dHJ migration or by mismatch repair bias towards the Landsberg allele during this DSB repair event. Biased repair towards the Landsberg allele is expected because the gRNA was designed to preferentially break the Columbia allele (Supplementary Table S1, see PAM polymorphism Chr3:1228466).

The evaluation of non-crossover (NCO) signatures in a hybrid background requires high coverage whole genome sequencing. Therefore, we have restricted our non-crossover identification only to plants with homozygous backgrounds at the target DNA DSB sites (at least 100,000 bp to each side of DNA DSB). Following screening of the 236 F2 plants (described above), 115 plants were selected. Out of these, 15 plants with non-crossover signatures were identified, corresponding to 12.9% of the total plants analyzed. These 15 NCO events originated from 5 out of the 13 sequenced F2 populations (Figure 2 and Supplementary Table S2). Most of these events occurred in F2 plants of target Chr3:1228466 (13/15 non-crossovers). The results show various sizes and patterns of NCO signatures with minimal conversion tract size ranging from 5 bp to 4349 bp (Figure 2). In some of the F2 populations few plants with similar (Figure 2c, plants #4 and #14 in F2 target Chr3:1228466) or complementary (Figure 2a, plants #10 and #20 in F2 target Chr3:1228466) NCO SNP distribution were observed, supporting the somatic origin of these events and their subsequent germinal transmission to the F2 plants. Target Chr3:4639826 which has features of heterochromatin embedded in the euchromatin region between the RFP and GFP markers, yielded two NCO events originating from two different plants out of 27 plants analyzed (Figure 2, Supplementary Table S2). Chr3:1854159 did not yield any IHR event out of 103 plants analyzed (Supp Table S2). Overall, the seed fluorescence markers did not enrich for CO events since a single CO event was found corresponding to the DSB site. Other crossover events between the seed markers, that we selected were apparently of meiotic origin and not CRISPR-Cas-related.

In Set#2, based on NHEJ rate, five plants from euchromatic target Chr3:1797873 (#4A, #4E, #4F, #5E, and #5F) and three plants from heterochromatic target Chr5:13564651 (including F1 plants #1A and #1C) were chosen for further analysis (Supplementary Table S3). F2 progenies of these plants, 217 in total, were sequenced using Illumina whole genome sequencing and processed as described above. Plants with induced crossover were not detected in this populations, but we were able to detect two F2 plants with targeted gene conversions; one (1 out of 140 F2 plants) in the euchromatic target Chr3:1797873 (Figure 2f) and the other (1 out of 77 F2 plants) at the heterochromatic target Chr5:13564651 (Figure 2g). F2 plant of Chr5: 13564651, a progeny of Chr5:13564651 plant #1d showed a short conversion tract of 22 bp or more, with an SNP pattern that correlates with F1 Columbia allele enrichment. In the euchromatic target (Chr3:1797873), we were able to detect one homozygote F2 plant with very long and a slightly interrupted conversion tract of ~7 Kb (Figure 2f). For this F2 plant to be homozygote, both maternal and paternal gametes should contain similar conversion tract. The chances that two long conversion tracts (>7 Kb) were created similarly during development in both gametes in meiosis are low, supporting the occurrence of a somatic gene-conversion event already in the F1 plant.

Overall, most of the non-crossover events show unique patterns of SNP distribution around the DSB site, probably resulting from independent non-crossover events (Figure 2). Five of the non-crossover plants contain short and simple tracts (all SNPs in the conversion tract coming from one allele) while ten plants showed longer and more complex conversion, with SNPs in the disrupted conversion tract originating from both alleles.

### 2.3. PacBio^®^ Sequencing for Somatic Recombination Analysis

To measure somatic recombination directly, and to test for its induction at the target site in F1 plants, we have sequenced 5 Kb amplicons of F1 plants from Set1# of gRNAs (Figure 1A, Supplementary Table S1). Genomic DNA was extracted from young floral buds (pre-meiotic tissues), upper leaves, and stems of these F1 plants, and three control plants of F1 Ler (WT) x Col tester (WT) served as a template for high-fidelity, long-range PCR amplification of 5 Kb fragments flanking each target. Amplicon libraries with PacBio^®^ barcoded adapters were built from these PCR products and sequenced with PacBio^®^ Sequel system. In this system, each molecule, corresponding to one of the possible alleles, is sequenced independently. Several SNPs along the 5 Kb fragments made possible the assignment of parental (or recombinant) alleles for each molecule sequenced.

**Figure 3:**
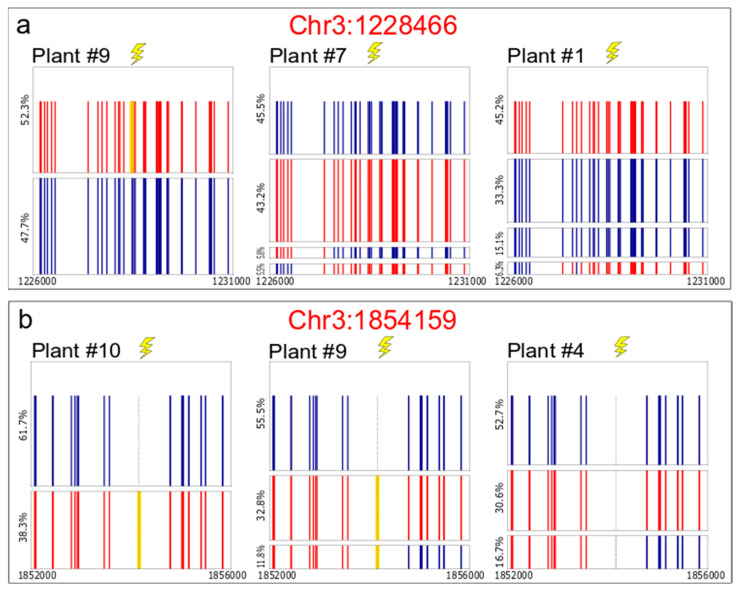
Somatic HR based repair- Distribution of parental, mutant, and recombinant alleles in Ler x Col background enables measurement and characterization of somatic DNA DSB repair events—allelic distribution resulting from somatic DSB repair, determined through PacBio^®^ sequencing of F1 plants from (**a**) the Ler x (Col Ubi:Cas9 x U6-26:Chr3:1228466), and (**b**) the Ler x (Col Ubi:Cas9 x U6-26:Chr3:1854159) populations. Every square represents the allelic distribution for each plant sequenced in a window of ~ 5 Kb flanking the DNA DSB target. In each square, each rectangle represents one consensus sequence calculated by the LAA2 analysis. The height of the rectangle represents the relative frequency of this consensus sequence/allele (percentage of subreads that aligned to calculate the consensus sequence). Red vertical lines represent Columbia SNPs, blue vertical lines represent Landsberg SNPs, the yellow line represents indels at the break site, and the dashed black line with lightning represent the DNA DSB site.

Sequencing and analyzing the allelic distribution of control plants (WT Ler x WT Col hybrids without DSB induction), enabled assessment of the advantages and limitations of this method. Five targets, out of eight tested, were selected for further analysis because they showed a balanced amplification of the parental alleles and no evidence for recombination products or recombination-like PCR artifacts in negative controls (Supplementary Figure S2). Sequencing of 5 Kb PCR fragments of F1 hybrid plants in populations that underwent DSB induction (Figure 1A) display a variety of allele repair patterns (Figure 3, Supplementary Figures S3–S7).

In two out of the five targets that were sequenced using PacBio^®^, Chr3:1228466 and Chr3:1854159, plants with both NHEJ and recombinant alleles were detected. The F1 hybrid plants with CRISPR-Cas9 targets Chr3:1261146, Chr3:1858597, and Chr3:4639823 (10/10, 2/2, and 5/5 plants, respectively, Supplementary Figures S5–S7) did not show any DNA DSB repair signature.

In the “hot” Chr3:1228466 target, three out of nine F1 hybrid plants with Ubi:Cas9 and gRNA, showed alleles with DNA DSB repair signatures (Figure 3a). From these, one plant contains an insertion of cytosine (+C) at the DNA DSB site, only in the Columbia allele (Figure 3a, plant#9). A second plant has two WT alleles of Columbia and Landsberg and two additional alleles, each of them presented in more than 5% of the reads, with reciprocal exchange of chromosomal segments at a distance of ~1–2 Kb from the DSB site (Figure 3a, plant#7). This reciprocal exchange may be the result of HR repair combined with migration of the conversion tract for a distance of ~1–2 Kb. The third plant contains the two WT parental alleles in addition to one allele with non-reciprocal exchange of chromosomal segments at a distance of ~600 bp–2 Kb from the DSB site (Figure 3a, plant#1), present in more than 6% of the reads. In this allele, the SNP pattern is not consecutive and Landsberg SNPs alternate with the Columbia SNPs. This kind of pattern may be explained by mismatch repair events along the conversion tract that might also have migrated at a distance of 600 bp–2 kb from the DNA DSB site. This pattern of somatic IHR is consistent with what was described above for germinal events.

Fourteen F1 hybrid plants derived from Ubi:Cas9 and the gRNA hot target Chr3:1854159 were sequenced and analyzed with the PacBio^®^ sequel system. Out of these, three plants contain the insertion of thymidine (+T) at the DNA DSB site only on the Columbia allele (Figure 3b plant #10 and Supplementary Figure S4, plants #10, #12, and #13). Two additional plants have the WT Landsberg allele, Columbia allele with thymidine insertion (+T), and an additional allele with both thymidine insertion and non-reciprocal exchange of chromosomal segments represented by 11.8% and 17.0% of the subreads (Figure 3b plant #9 and Supplementary Figure S4, plant #6 and #9), respectively. Interestingly, these five plants had a similar NHEJ pattern and came from the same cross of parents, reinforcing the hypothesis that the NHEJ event occurred in the previous generation and was inherited by these F1 hybrid plants. In addition, three plants contained the WT Landsberg allele, WT Columbia allele, and an additional allele with a non-reciprocal exchange of chromosomal segments represented by 13.8%, 14.1%, and 16.7% of the subreads (Figure 3b, plant#4, Supplementary Figure S4, plants #2, #3, and #4), respectively. Due to the limited length of the PacBio^®^ sequenced amplicon (5 Kb) it is hard to determine if the somatic HR events we documented are crossover events or non-crossover events with long conversion tracts.

## 3. Discussion

Previous studies have used transgenic markers to analyze DSB-induced somatic intrachromosomal HR based repair [41,42], inter-chromatid unequal crossover [21], or inter-homolog recombination in plants [43]. In recent works [23,44], we demonstrated that DSB repair can also occur via HR between homologous chromosomes in somatic cells at a single endogenous *locus*, the *Psy1* and the *CRTISO* genes in tomato. Here, we extended the study of targeted IHR to several targets in *Arabidopsis thaliana*, in various epigenetic contexts such as euchromatin, heterochromatin islands embedded within euchromatin to heterochromatin located at pericentric loci. We found that Cas9-mediated DSBs can lead to targeted IHR in all the different chromatin types studied, with NCO as the most frequent IHR repair outcome. We discuss the repair patterns and frequencies at these loci.

### 3.1. IHR Frequency

Overall, out of 26 targets analyzed, only 14 showed evidence of DSB induction, as seen from NHEJ-mediated indels, and out of these 14, only four gave rise to germinal transmitted IHR events. In total, we found 17 targeted NCO events and one single CO out of 453 F2 plants analyzed. There was a great variability between gRNAs in their ability to induce IHR-repair; one particular target (Chr3:1228466) gave rise to 13 out of the 17 targeted NCO events. This same target gave rise to the single targeted CO event. In other words, the rate of NCO per F2 plant analyzed ranged between 12% (13/106 F2 plants) for target Chr3:1228466 and ~7% (2/27 F2 plants) for target Chr3:4639826, and ~1% for targets Chr3:1797873 (1/140 F2 plants) and Chr5:13564651 (1/77 plants). The CO rate was much lower: for target Chr3:1228466 we had one event out of 106 plants analyzed (=0.9%) and none for the other loci. What determines the ability of DSBs at different targets to be repaired by IHR is not clear at this point. This might be due to differences in the availability of the homologous template or in the accessibility of the IHR repair complex to the targets. This might be affected by the 3D organization of chromosomes or other unknown features.

This strong bias in favor of NCO compared to CO events might be due to the HR machinery at work in somatic cells. Pathways such as SDSA, that give rise only to NCO events, might be more prominent in somatic tissues where DSBs were induced, compared to recombination pathways that can give rise to COs, such as pathways generating dHJ, or HJ-like structures, as with ClassII COs (Non interfering/Mus81 dependent CO). Interestingly, while in natural meiotic recombination NCO events seem to be rare compared to CO events (see [5] for review), with Cas9-induced IHR as reported here it seems to be the other way around. This might reflect a difference between somatic and meiotic HR.

### 3.2. Conversion Tract Patterns

The length of conversion tracts was variable, ranging from 5 pb to 7505 bp, with short and simple conversion tracts or longer and more complex tracts (Figure 2). The phenomena of a long and complex conversion tract (also known as an interrupted or discontinued gene conversion tract) was also observed in our previous work on tomato *Psy1* [23] and *CRTISO* [44] and was also shown for both somatic and meiotic repair of yeast [45,46] and mammalian cells [47,48]. Complex conversion tracts were shown to be the result of the mismatch repair mechanism in heterozygous heteroduplexes of D-loop in SDSA or double Holliday Junctions intermediates [49,50,51,52]. A recent study in yeast suggests an additional mechanism that explains complex conversion tracts, called multi-invasions (MIs). With this mechanism, the ssDNA filament invades more than one dsDNA donor and synthesizes its sequence based on these two donors [53]. The two donors can be unbroken and broken chromosomes or homologous chromosome and unbroken sister chromatid, in case that DNA DSB occurred after DNA replication. Such examples of multiple template switches, or complex conversion tracts were also shown in plants [54,55], but not between homologous chromosomes.

### 3.3. Somatic IHR

Along this study Cas9 was expressed under the constitutive Ubiquitin promoter of parsley that was shown to be active in somatic tissue [14] but has not been tested in meiotic tissue. Therefore, we cannot rule out that DSBs were also induced in meiotic cells. Nevertheless, several results indicate that somatic IHR took place in this study. For example, the presence of different Arabidopsis F2 plants with similar non-crossover conversion tracts (Figure 2c plants #4 and #14), coming from the same F1 plant, reinforces the assumption that this repair is somatic. A second result supporting somatic IHR is the documentation of long homozygote conversion tract in F2 plant of Chr3: 1797873, suggesting it was formed before meiosis and became germinally transmitted through both male and female lineages (Figure 2f). In addition, PacBio^®^ sequencing of F1 plants showed relatively high levels of somatic IHR repair (ranging from 6.3% to 17%, with an average of 13%, Supplementary Figures S3 and S4), in agreement with the 14% IHR rate we previously documented in the tomato *Psy1* allele [23]. The occurrence of somatic IHR in Arabidopsis is not trivial considering that there is no evidence of somatic pairing in this species [56]. One hypothesis to consider for future research is that somatic pairing is induced when a DSB is formed. Surprisingly, there was no good correlation between the somatic events measured and sequenced by PacBio^®^ and those that were germinally transmitted as determined by whole genome sequencing in F2. This may be due to chimerism in F1 repair events or due to F1 tissue sampling method. Another possible explanation is that the frequent endopolyploidy in Arabidopsis somatic tissues, including the floral buds, stalks, and floral leaves we have sampled [57], may lead to relatively high levels of somatic IHR that is not transmitted to the next generation.

### 3.4. IHR in Euchromatin versus Heterochromatin

Our finding of Cas9-induced IHR for both euchromatic and heterochromatic loci constitutes a significant difference with classical meiotic recombination. During meiosis, SPO11 induces DSBs preferentially in low methylated DNA marked with H3K4me3 nucleosomes [34,58], shifting crossover events to genomic areas containing these features, namely euchromatin [25,31,59]. Conversely, Cas9 was shown to be less sensitive to DNA methylation [60]. This might explain why we could obtain NCO events in diverse types of chromatin, including in pericentric regions (Figure 2g) that are often recombination deserts in higher plants [25,26,61]. To our knowledge this is the first work that shows heterochromatic and pericentric targeted IHR repair in plants. While there might be differences in the efficiency of Cas9 DSB induction in euchromatin versus heterochromatin (6/7 euchromatic targets showed NHEJ based repair vs. 2/7 in heterochromatic pericentric targets, Supplementary Table S1 Set#2) it seems that there are no fundamental differences in the mode of repair, where both NHEJ and IHR can be used. Out of the four gRNAs that gave rise to IHR events, two were in euchromatin (Chr3:1228466 and Chr3:1797873) one was in heterochromatin embedded in euchromatin (Chr3:4639826), and one in pericentric heterochromatin (Chr5:13564651). While these are small numbers, they show the same trend as a recent study of hybrid mice cells coming from F1 individuals with hypo-methylated father and hyper-methylated mother, showing that DNA methylation delays the repair of Cas9 induced DSBs but does not affect the end-product (NHEJ signatures or NHEJ vs. HDR ratio) of repair [62]. Similarly, in *Drosophila melanogaster*, live imaging and sequence analysis of an I-SceI induced single DSB in both euchromatic and heterochromatic loci showed similar kinetics of NHEJ and IHR based repair [63]. Altogether, these experiments in different species build a consistent view that DSB induction is the main cause for differences in recombination between euchromatin and heterochromatin. This might suggest that during meiotic recombination, heterochromatin is “cold” due to SPO-11′s ability to mediate DSBs in these regions, rather than due to the repair machinery (NHEJ vs. HR).

To conclude, this study discovers several new features of targeted IHR events, such as the prominence of NCO compared to CO repair products, the length and patterns of conversion tracts and the possibility of targeting IHR, not only to euchromatin, but also to heterochromatin regions, suggesting that DSB induction is the bottleneck for HR in heterochromatin. This work opens several new avenues of future research to better understand the CO vs. NCO repair pathway choice, the mechanism of induced somatic pairing, the differences between somatic and meiotic recombination, etc. It also opens new prospects for precise breeding technologies via chromosome engineering in both euchromatin and heterochromatin. In particular, targeted gene conversion would eliminate the necessity of multiple back-crosses in order to remove undesired introgressions. Targeted CO efficiency would have to be increased to become a useful tool. An alternative for precise breeding via chromosome engineering being the induction of reciprocal translocation, using NHEJ, which yields similar outcomes as CO [64].

## 4. Materials and Methods

### 4.1. Plant Material

All Arabidopsis plants were germinated on ½ MS plates and transmitted to soil at the age of one week post germination. Columbia tester line plants were built as described by Melamed-Bessudo et al. [38]. Transgenic plants were grown in ½ MS plates with 50 mg/L kanamycin and/or 20 mg/L hygromycin. Plant grew in growth chambers at a temperature of 20–22 °C under short day conditions (8 h light, 16 h dark) for 4 weeks and were then transmitted to long day (16 h light, 8 h dark). F1 plants of targeted IHR in euchromatic regions vs. pericentric heterochromatic regions experiment were treated with 4 cycles of 30 °C for 24 h at the age of 4 weeks.

### 4.2. Plasmids and Plant Transformation

Throughout this work we have used *Streptococcus pyogenes* Cas9 (spCas9), codon optimized to *A. thaliana*, kindly provided us by the Holger Puchta’s lab at Karlsruhe Institute of Technology, Germany [14]. The Cas9 was expressed under parsley Ubiquitin promotor, UBQ4 (referred as Ubi) and Pea3A terminator. The gRNAs were expressed under the Arabidopsis U6-26 promoter [65] with terminator of seven thymidine. The kanamycin resistance gene was expressed under the *Nopaline Synthase* (Nos) promotor with Nos terminator, referred as Nos:NptII, and hygromycin resistance was expressed under two copies of the constitutive Cauliflower mosaic virus 35S promoter with Nos terminator, referred as 35SX2:Hpt.

All plasmids used for Arabidopsis transformation were cloned using the GoldenBraid cloning system [66]. Arabidopsis plants were transformed by *Agrobacterium tumefactions* GV3101 with floral dip transformation [67].

In set #1 Ubi:spCas9 and each U626:gRNA were transformed separately to Arabidopsis Columbia tester lines (Col3-4/20) and WT Columbia, respectively, and then plants were crossed and selected with both kanamycin and hygromycin. The selected plants were crossed to Ler in order to generate heterozygous F1 hybrids, maintaining the selection to both antibiotics.

For set #2, each of the 14 gRNAs was cloned separately into a kanamycin resistant vector with Ubi:spCas9 U626:gRNA and then transformed to Arabidopsis Columbia tester lines (Col3-4/20). The positive kanamycin resistant T1 plants were crossed with WT Arabidopsis Landsberg ecotype plants in order to generate heterozygous F1 hybrids. F1 seeds were sown on kanamycin selection media in order to select for plants expressing the spCas9 and gRNA.

### 4.3. DNA Amplification and Sequencing

DNA amplicon samples for high-throughput sequencing were amplified using Phusion^®^ High-Fidelity DNA polymerase (Waltham, Massachusetts, USA) and 18 PCR cycles (for specific primers of each experiment see primers list at Supplementary Table S4). Libraries were prepared as Blecher-Gonen et al. [68]. For whole genome sequencing Arabidopsis samples, DNA was purified from rosette leaves of F2 plants using a DNA purification kit (MACHEREY-NAGEL^®^) and then 300 ng sheared by sonication to 200–500 bp. A total of 10 ng of fragmented DNA per plant was used for libraries preparation, as described by Blecher-Gonen et al. [68]. High-throughput Sequencing was performed at the life sciences core facilities unit at the Weizmann Institute of Science with the Illumina HiSeq 2500 platform, NextSeq, or NovaSeq; all of them for 2 × 150 paired end reads.

For NHEJ F1 plant, DNA was extracted from somatic tissue of cauline leaves. In order to evaluate the relative repair rates and footprints via the NHEJ repair pathway, 300 bp amplicons flanking the break site were amplified and sequenced from ten independent F1 and three control plants from each target, followed by analysis using NGS Cas-Analyzer [37].

DNA samples for PacBio^®^ sequencing were extracted from upper leaves, stems, and young buds of F1 Arabidopsis plants as described by Fulton et al. [69]. The 5 kb amplicons flanking the DSB site were amplified using TaKaRa LA Taq^®^ DNA polymerase Hot-Start version (for primers-see primer list at Supplementary Table S5). Libraries preparation was conducted according to PacBio^®^ barcoded adapters protocol and sequence at the Weizmann Institute Life Sciences Core facilities unit using PacBio^®^ sequel system.

### 4.4. Sequence Analysis

Whole genome sequencing reads of Arabidopsis F2 were aligned to the TAIR10 version of the Arabidopsis genome (Columbia reference genome) using BWA and Samtools and then viewed and documented using IGV browser [40]. Crossovers were detected using costume made script, based on hidden markov model, and available at the following Github address—https://github.com/zisserj/coda (accedded on 7 November 2021).

PacBio^®^ reads of Arabidopsis F1 were analyzed using SMRT link version 1.0. Sequencing products were first sorted into amplicon libraries (each library originated from a different plant) using SMRT^®^ Link Barcoding analysis, and then consensus sequences were calculated for each plant using SMRT^®^ Link Long Amplicon Analysis (LAA2). These two steps were conducted without any reference sequence and, hence, the consensus sequences are not biased towards any of the ecotypes (Columbia or Landsberg). Each of these ~5 Kb consensus sequences were aligned to *A. thaliana* genome (TAIR10-Columbia ecotype reference) using BWA MEM algorithm and then indexed using SAMTOOLS algorithm. SNP calling and graphs were generated using python scripts.

## Supplementary Materials

The following are available online at https://www.mdpi.com/article/10.3390/ijms222212096/s1.
