# Supplementary material for: Targeted Inter-Homologs Recombination in Arabidopsis Euchromatin and Heterochromatin
Source: Int J Mol Sci. 2021 Nov 9;22(22):12096. doi: 10.3390/ijms222212096 (PMC8622013; doi:10.3390/ijms222212096)
Supplement: Supplementary file 1 [file ijms-22-12096-s001.zip › ijms-1424565-SI.pdf]

**Supplementary Materials:**

**Table S1: Targets Description.** Targets names described the chromosome number and position. The epigenetic selection of the targets was based on CpG methylation and Nucleosome occupancy data from Zemach et al. 2013 [71], H3K4me3 data was based on Roudier et al. 2011 [36]. Set#1 yellow and blue are highlighting euchromatic and heterochromatic targets embedded within euchromatin respectively. Set #2 yellow and blue highlighting are euchromatic targets on chromosome arms and heterochromatic pericentric targets respectively.

| Cas9 DSB site | Cas9 Target Seq. (PAM in red) | Col vs Ler | Gene | Cpg methylation (0.0-1.0) | Nucleosomes occupancy (0 -296) | H3k4me3 (-4.85-5.05) | Heterochromatin/ Euchromatin | %NHEJ |
|---|---|---|---|---|---|---|---|---|
| **Set#1** | | | | | | | | |
| Chr3:1228466 | aactgctttgaatgtccatatgg | tgg-->tag | at3g04560.1 | 0 | 1.95 | 0 | Euchromatin | 4.4 |
| Chr3:1222177 | gctggagaaccgccgtttaacgg | similar | at3g04530.1 | 0 | 7.82 | 2.77 | Euchromatin | 19.8 |
| Chr3:1261146 | cgcttgaatgatgaccactgcgg | similar | at3g04640.1 | 0 | 74.13-96.53 | 2.25 | Euchromatin | 0 |
| Chr3:1352618 | atattgtttttcatattttttgg | tgg-->ttg | — | 0 | 1.02 | 0 | Euchromatin | 0 |
| Chr3:1352127 | ccaaaaaaaaaaaatacagtcgt | similar | — | 0 | 5.49 | 0 | Euchromatin | 0 |
| Chr3:1854159 | gtttccgccaccaccgcctccgg | similar | at3g06130.2 | 0 | 27.25 | 3.27 | Euchromatin | 11.6 |
| Chr3:1843852 | tctacaaagtcattgaaggttgg | ggg-->agg | at3g06110.3 | 0 | 4.3 | 0 | Euchromatin | 0 |
| Chr3:1858597 | agagttgatctgtggctgtggcgg | similar | at3g06140.1 | 0 | 13.95 | 3.04 | Euchromatin | 73.6 |
| Chr3:4684724 | tgactgcaggtgagcttacacgg | similar | at3g14120.3 | 0.81 | 2573 | -2.15 | Heterochromatin | 37.7 |
| Chr3:1565357 | ccttggaaattttctcttcccaa | cct-->ctt | at3g05420.1 | 1 | 36.04 | -0.78 | Heterochromatin | 0 |
| Chr3:1559196 | ccccgacatttaatgatgttttt | 5 kb del in Ler | at3g05415.1 | 0.85 | 138.175 | 0 | Heterochromatin | 0 |
| Chr3:4639826 | agaagttcagaaagtcgcccagg | similar | at3g14010.4 | 1 | 65.74 | -2.47 | Heterochromatin | 0.8 |
| **Set#2** | | | | | | | | |
| Chr3:1231856 | ttgttggaggaagcgtggtgggg | ggc-->ggg | at3g04570.1 | 0 | 13.1 | 1.23 | Euchromatin | 1.3 |
| Chr3:1265118 | ccagatctacggtactcattttc | tca-->cca | at3g04660.1 | 0 | 21.26 | 0.79 | Euchromatin | 4.8 |
| Chr3:1797873 | cctcacaatgattccagccgttt | tct-->cct | at3g05990.1 | 0 | 9.42 | 0.76 | Euchromatin | 35.8 |
| Chr3:2036584 | atcaacgccccaagtgcgttagg | aga-->agg | at3g06540.1 | 0 | 20.88 | 3.1 | Euchromatin | 6.8 |
| Chr3:4064382 | cccagcagcggcgttaacaagaa | gcc-->ccc | at3g12800.1 | 0 | 11.83 | 2.96 | Euchromatin | 0.4 |
| Chr5:18158030 | gtagcgtatacgaatataatggg | gga-->ggg | at5g44973.1 | 0 | 3.02 | -1.2 | Euchromatin | 9.1 |
| Chr5:19187725 | cccgagcactgggatgcctattg | acc-->ccc | at5g47250.1 | 0 | 6.34 | 1.825 | Euchromatin | 0 |
| Chr1:12765381 | ccttaaggaccatgttaaggcgt | tct-->cct | at1g34790.1 | 0.6 | 30.16 | -1.03 | Heterochromatin | 0 |
| Chr1:13325352 | cccatctccaacagattagaagg | tcc-->ccc | at1g35840.1 | 0.933 | 39.82 | -2.94 | Heterochromatin | 0 |
| Chr1:13394390 | ccaacccacgtgaacggatcccc | tca-->cca | at1g35960.1 | 0.6875 | 36.45 | -3.4 | Heterochromatin | 0 |
| Chr1:16855554 | agaagactggctctgttcacggg | gga-->ggg | at1g44510.1 | 0.833 | 42 | -1.61 | Heterochromatin | 0 |
| Chr5:13564651 | gtgagacattcccgtgaggacgg | cga-->cgg | at5g35340 | 0.81 | 19.5 | -0.629 | Heterochromatin | 13.9 |
| Chr5:15815729 | ccctgcctcagaggaagtggttt | tcc-->ccc | at5g39500.1 | 1 | 19.52 | 1.8 | Heterochromatin | 0 |
| Chr5:16450556 | ccagagcggactaattagaggta | tca-->cca | at5g41100.2 | 0.56 | 22.72 | -0.88 | Heterochromatin | 11.7 |

**Table S2: Germinaly transmitted DNA DSBs events in F2 Arabidopsis plant populations, Set #1: Cold and Hot spots.** DSB signatures documented in thirteen F2 plant populations by whole genome sequencing are summarized in the table.

| F1 plant | F2 seed sorting (GFP only/RFP only/ no sorting) | Total F2 plants sequenced | Total Cas9+, gRNA+ plants (% in the population) | Plants with NHEJ signature (% of plants in F2 population, % alleles in F1 population- average of PacBio and Illumina data) | Plants with visible Induced crossover (% of plants in F2 population) | Plants with visible Induced non-crossover (% of homozygote plants in F2 population) | Homozygote plants at DSB area | Heterochromatin/ Euchromatin |
|---|---|---|---|---|---|---|---|---|
| Chr3:1228466-#1 | No sorting | 35 | 18 (51%) | 0 (0%, 0%) | 0 (0%) | 4 (20%) | 20 | Euchromatin |
| Chr3:1228466-#2 | RFP only | 17 | 9 (53%) | 0 (0%, 0%) | 1 (5.88%) | 2 (22.22%) | 9 | Euchromatin |
| Chr3:1228466-#2 | GFP only | 10 | 1 (10%) | 0 (0%, 0%) | 0 (0%) | 0 (0%) | 5 | Euchromatin |
| Chr3:1228466-#5 | RFP only | 6 | 1 (17%) | 0 (0%, 0%) | 0 (0%) | 3 (60%) | 5 | Euchromatin |
| Chr3:1228466-#5 | GFP only | 4 | 4 (100%) | 0 (0%, 0%) | 0 (0%) | 0 (0%) | 1 | Euchromatin |
| Chr3:1228466-#7 | No sorting | 34 | 10 (29%) | 0 (0%, 0%) | 0 (0%) | 4 (22.2%) | 18 | Euchromatin |
| Chr3:1854159-#2 | No sorting | 39 | 17 (44%0 | 1 (3%, 0%) | 0 (0%) | 0 (0%) | 19 | Euchromatin |
| Chr3:1854159-#6 | No sorting | 25 | 8 (32%) | 12 (48%, 50%) | 0 (0%) | 0 (0%) | 10 | Euchromatin |
| Chr3:1854159--#9 | GFP only | 14 | 9 (64%) | 0 (0%, 48%) | 0 (0%) | 0 (0%) | 4 | Euchromatin |
| Chr3:1854159-#11 | GFP only | 5 | 0 (0%) | 0 (0%, 0%) | 0 (0%) | 0 (0%) | 3 | Euchromatin |
| Chr3:1854159-#12 | GFP only | 10 | 2 (20%) | 4 (40%, 50%) | 0 (0%) | 0 (0%) | 3 | Euchromatin |
| Chr3:1854159-#13 | GFP only | 10 | 2 (20%) | 2 (20%, 47%) | 0 (0%) | 0 (0%) | 3 | Euchromatin |
| Chr3:4639826-#4 | No sorting | 27 | 4 (15%) | 0 (0%, 0%) | 0 (0%) | 2 (12.5%) | 16 | Heterochromatin |
| Total | | 236 | 85 (36%) | 19 | 1 (0.42%) | 15 (12.9%) | 115 | |

**Table S3: Germinal transmitted DNA DSBs events in F2 Arabidopsis plant populations. Set#2: Euchromatic and Heterochromatic plants. DSB signatures documented in eight F2 plant populations by whole genome sequencing are summarized in the table.**

| F1 plant | Total F2 plants sequenced | Plants with NHEJ signature, based on F2 WGS (% of plants in F2 population) | Plants with visible Induced non-crossover (% of plants in F2 | Heterochromatin/ Euchromatin |
|---|---|---|---|---|
| **Chr5:13564651-#1A** | 13 | 2 (15.4%) | 0 (0%) | Heterochromatin |
| **Chr5:13564651-#1C** | 19 | 4 (21.1%) | 0 (0%) | Heterochromatin |
| **Chr5:13564651-#1D** | 45 | 11 (24.4%0 | 1 (2.2%) | Heterochromatin |
| **Chr3:1797873-#4A** | 20 | 4 (20%) | 0 (0%) | Euchromatin |
| **Chr3:1797873-#4E** | 19 | 8 (42.1%) | 1 (5.2%) | Euchromatin |
| **Chr3:1797873-#4F** | 19 | 3 (15.8%) | 0 (0%) | Euchromatin |
| **Chr3:1797873-#5E** | 46 | 2 (4.3%) | 0 (0%) | Euchromatin |
| **Chr3:1797873-#5F** | 36 | 2 (5.6%) | 0 (0%) | Euchromatin |
| **Total** | 217 | 36 (16.6%) | 2 (1%) | |

**Table S4: List of Primers for NHEJ experiments.**

| | |
|---|---|
| **Primers for NHEJ sequencing-Set#1** | |
| 4639826_HT_F | NNNNNNAATCTGAAAGCTCTTTCGGG |
| 4639826_HT_R | NNNNNNCGATTGGAGATTGAGGTGGT |
| 4684724_HT_F | NNNNNNAAAACAAGATGTTACAATGAAAGC[illegible] |
| 4684724_HT_R | NNNNNNTCGAGATACATATGTCTGCAATGT[illegible] |
| 1858597_HT_F | NNNNNNCTGAGGGTGATAATGGTAATGG |
| 1858597_HT_R | NNNNNNTTGTGCTTTCTGTCTTGTCTCTG |
| 1565357_HT_F | NNNNNNTGGCGGTGATAACAAGAGTG |
| 1565357_HT_R | NNNNNNTGAAAAGGCTTCTACCGTGTT |
| 1559196_HT_F | NNNNNNGTTAAAACCGCGCACATTC |
| 1559196_HT_R | NNNNNNTGAGTTCCCCACAATCTCCT |
| 1228466_HT_F | NNNNNNAAAGCTGTCATCAGTGAAGAGGT |
| 1228466_HT_R | NNNNNNCCTCGAAGCTTGCTATACGG |
| 1222177_HT_F | NNNNNNCGCTGGTGTAGTGATATACACG |
| 1222177_HT_R | NNNNNNTTTCCAACGTTCATCATCCA |
| 1843852_HT_F | NNNNNNTTGGCTCCTCCTTATCCTGA |
| 1843852_HT_R | NNNNNNCCATGAAGCAATGAACCAAA |
| 1261146_HT_F | NNNNNNAAACCAAACCTAAACCGGCTA |
| 1261146_HT_R | NNNNNNTCGAAGCCAATTTACCGAAA |
| 1352127_HT_F | [illegible]NNNNNTTTACATATTTTAATTAGATTGGTAGAACAA[illegible] |
| 1352127_HT_R | NNNNNNTGCCTCGTTGTCACTTAACG |
| 1352618_HT_F | NNNNNNAGTTCTTTTGGCAATCACGTT |
| 1352618_HT_R | NNNNNNTTGGCCTTTTCCTCTCACAC |
| 1854159_HT_F | NNNNNNCGTTTTTCTGGCCTTTCTTG |
| 1854159_HT_R | NNNNNNTCAGGTGTTTTCACGACCAA |
| **Primers for NHEJ sequencing-Set#2** | |
| il3_1856F | TAACCGGTTCTTTCTTGCCAGG |
| il1856R | CCGTCGCCTCCACCAGCAC |
| il5118F | AGTCACCGCCGAAATCCATAG |
| il5118R | ATCATAGCTTTAGTATTGTTTTGAC |
| il7873F | GATTAGCTTATGAGTCTCTGTTC |
| il7873R | GGGAGTTTCAGGTTCGCTAGC |
| il6584F | TACATAGATACATCCTTTGACAGC |
| il6584R | CTCAAGACCAAAGAGGGGATAG |
| il4382F | ATGAGAAAACGAAAAAGGAACCTG |
| il4382R | TGCTCTTCGATCTCTTGGAATC |
| il8030F | CTCTGATCTGTAGTTTATTTCAAG |
| il8030R | ATCATCCAACATTCAAATACAGAC |
| il7725F | TGTCCTCATTTGCCCTCATAAC |
| il7725R | GGTTGAATCGTCTAAAGATGCAG |
| il5352F | AGAAATCTCAACAAATCTCCAACC |
| il5352R | TCACAGTCTTGTATTCTTCTAGC |
| il4390F | AGACCAAGCTGAAATAATGAAGG |
| il4390R | ACAGAGCGTTATCATTTATGGGA |
| il5729F | TTACTCTTGAAACTGTGAATGTGG |
| il5729R | CATCTAATCAACTCATGCATTGTG |
| il0556F | CGCCTCTTCCGCTGACTCCA |
| il0556R | CTTCTATATTTACTTATGGGTCTC |
| il5381F | TTGACCATCCTCGTTCCAAGC |
| il5381R | AGACAGATGAATTAGAAGCCTGC |
| il5554F | CAGGAGAAGACGAGGAGGATG |
| il5554R | TCAGCGTCTTCGTGTGTTTGG |
| il4651F | TCGAAAGGTGATGCAAAGATAGAG |
| il4651R | ATCTACACATTCACAATCACGCAC |

**Table S5: List of Primers for PacBio® experiments.**

| Primers for PacBio sequencing | |
|---|---|
| pb_1222177_f | TCCATCGTACCGTCTTCCTC |
| pb_1222177_r | CGATTTGGGATTTCGACCTA |
| pb_1228466_f | CTACCAACCGTTCCGACCAC |
| pb_1228466_r | GGAACCAGCAGGACGTCCAC |
| pb_1261146_f | TTTGTTCTGGATAAAAAGACAATTC |
| pb_1261146_r | TCCACACCTTGAAGCATCCCG |
| pb_1261146_f | ACAGACCAAAATCAGACCCTTC |
| pb_1261146_r | TTATTGTTTTTGTTCTTCTCTGACA |
| pb_1565357_f | CAAACCATGGGAACTATTACAC |
| pb_1565357_r | CCTTCTACCTACTTCATTCATAG |
| pb_1843852_f | ATCCTCCTGAAATCGGTCTAAC |
| pb_1843852_r | TATCTGGGATCCTATTAAATAAGC |
| pb_1854159_f | GTAATAATTGCATGGTTGTACGT |
| pb_1854159_r | GAGAGAAAAAGAGGAGAAGTTGA |
| pb_1858597_f | ATCTTTGGCGCATTACTGTTAC |
| pb_1858597_r | TGTAGAGTTGACTTTTGAGTAGCAT |
| pb_4639826_f | CCCGTTCCGAATTTGTCAGGA |
| pb_4639826_r | TAAAATAGAAAGTAAAGATTCAGAC |
| pb_4684724_f | TGGTGGTAGGGGTCTCGAAAT |
| pb_4684724_r | CTATCTATGTTAGGAGAAATCGC |

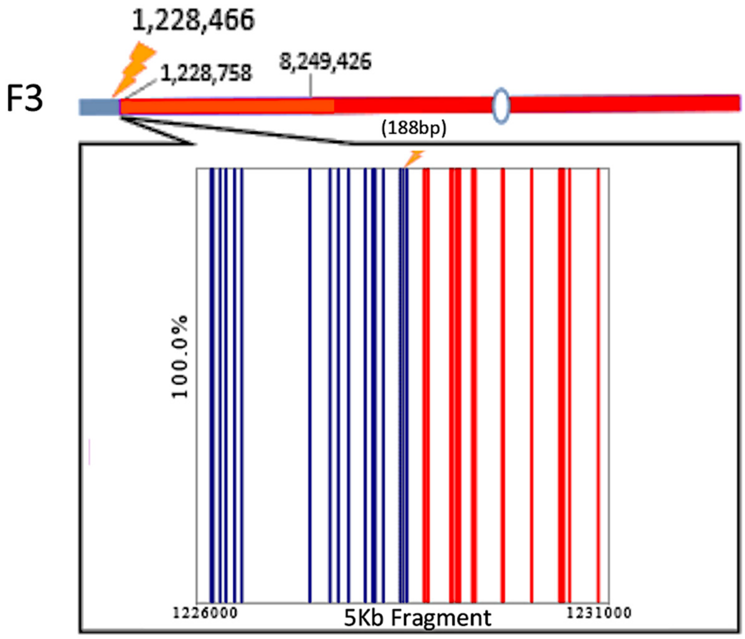


**Figure S1: Confirmation of the crossover site with PacBio® sequencing.**

A 5Kb fragment flanking the DSB site was amplified from the homozygous F3 offspring Chr3:1228466_RFP only plant# 14 and sequence with PacBio® Sequel system. The allelic distribution shown in the square. Red vertical lines represent Columbia SNPs, blue vertical lines represent Landsberg SNPs. The height of the rectangle represents the frequency of this consensus sequence/allele (percentage of subreads that aligned to calculate the consensus sequence); >2000 single molecules were sequenced. The distance between the induced DNA DSB site (yellow lightning) and the crossover site was 188bp.

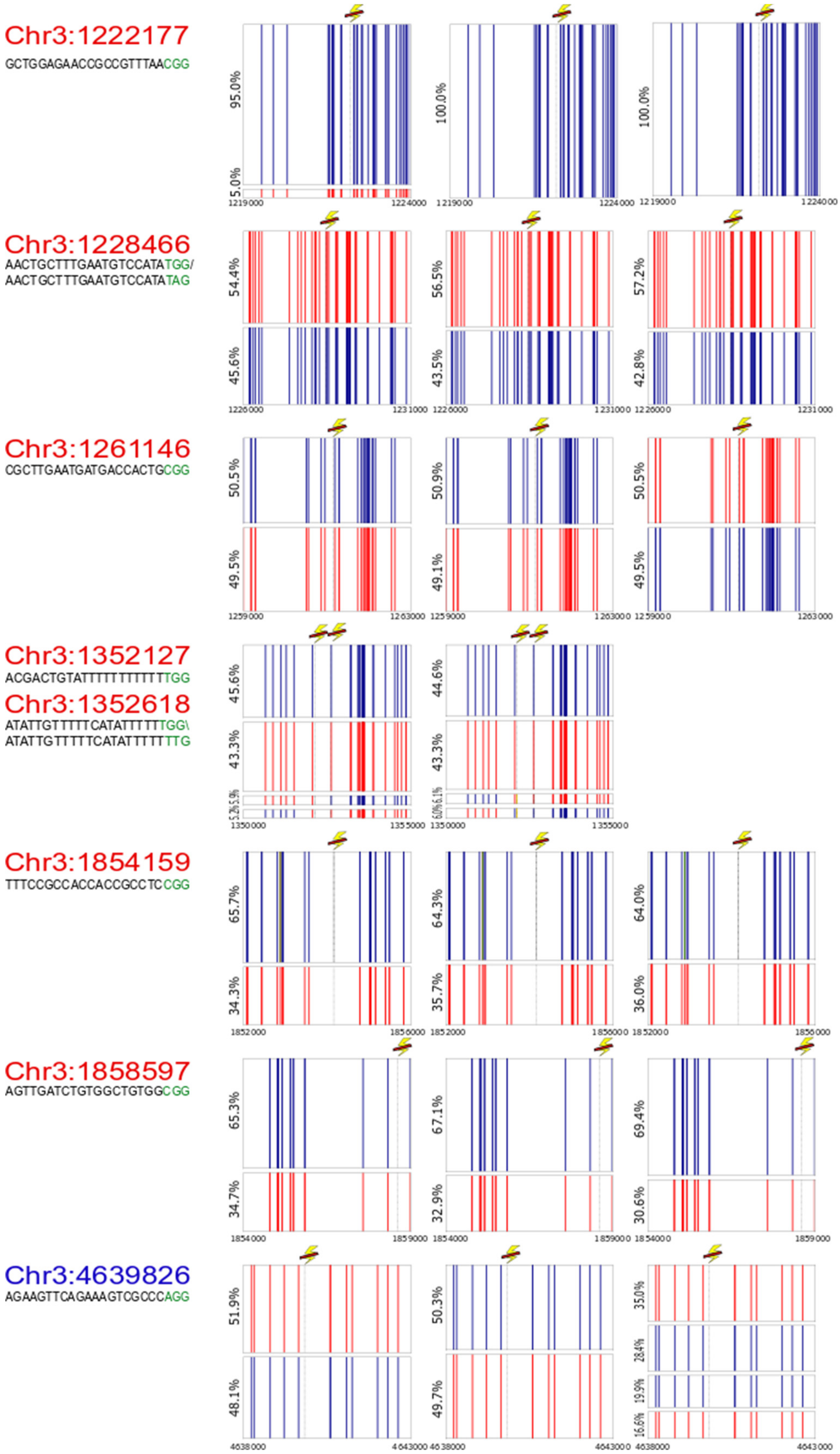
Chr3:1222177
GCTGGAGAACCGCCGTTTAACGG
95.0%
5.0%
1219000
1224000
100.0%
1219000
1224000
100.0%
1219000
1224000
Chr3:1228466
AACTGCTTTGAATGTCCATATGG/
AACTGCTTTGAATGTCCATATAG
54.4%
45.6%
1226000
1231000
56.5%
43.5%
1226000
1231000
57.2%
42.8%
1226000
1231000
Chr3:1261146
CGCTTGAATGATGACCACTGCGG
50.5%
49.5%
1259000
1263000
50.9%
49.1%
1259000
1263000
50.5%
49.5%
1259000
1263000
Chr3:1352127
ACGACTGTATTTTTTTTTTTTTTGG
Chr3:1352618
ATATTGTTTTTCATATTTTTTGG\
ATATTGTTTTTCATATTTTTTTG
45.6%
43.3%
1350000
1355000
44.6%
43.3%
1350000
1355000
Chr3:1854159
TTTCCGCCACCACCGCCTCCGG
65.7%
34.3%
1852000
1856000
64.3%
35.7%
1852000
1856000
64.0%
36.0%
1852000
1856000
Chr3:1858597
AGTTGATCTGTGGCTGTGGCGG
65.3%
34.7%
1854000
1859000
67.1%
32.9%
1854000
1859000
69.4%
30.6%
1854000
1859000
Chr3:4639826
AGAAGTTCAGAAAGTCGCCCAGG
51.9%
48.1%
4638000
4643000
50.3%
49.7%
4638000
4643000
35.0%
28.4%
19.9%
16.6%
4638000
4643000

**Figure S2: Allelic distribution of Ler and Col alleles in Control plants (with No DSB induction) as shown by long-range sequencing.** Distribution of parental alleles in 3 control plants of F1 Ler x Col tester line at different targets using PacBio® sequencing (target# and sequences are shown on the left of each row of squares PAM sequences are green). Within every square we show the allelic distribution for one plant in a~5Kb window flanking the target. The red and blue lines correspond to the Columbia and Landsberg SNPs respectively, dashed black line with lightning represent the DNA DSB site (the sites are signed by X because there is no DSB induction in the control plants). In each square, each rectangle represents one consensus sequence. In some cases, (for example in cold target – chr3:4639826), there are more than two consensus sequences of each allele, differing in indels that are not located at the DSB point and correspond to neither Col/Ler SNPs. The height of the rectangle represents the frequency of this consensus sequence/allele. In case of the presence of a SNP in the target or PAM site (e.g., target chr3:1352618) the Columbia allele sequence is before the slash and Landsberg allele is after the slash.

Targets Chr3:1261146, Chr3:1228466 and Chr3:4639826 show balanced (~1:1) allelic distribution of Columbia vs. Landsberg (~ 50% of amplification of the Col allele and ~50% of the Ler allele). For targets 1854159 and 1858597 the allelic distribution is biased toward the Ler allele with 31-36% of molecules coming from Col allele and 64-71% coming from Ler allele, possibly due to differences in PCR efficiencies for each allele. Target 1222177 also shows biased allelic distribution, with 0-5% of amplification of the Col allele and 95-100% of the Ler allele. For all the above targets, in the control plants without DSB induction no recombinant molecules were obtained among the amplification products. For targets 1352127 & 1352618 (shown in the same sequence window due to targets proximity), the allelic distribution is balanced, both control plants showed alleles with recombinant consensus sequences. For this target we also observed a deletion of -A at the DNA DSB site 1352127 in control plant #2. The deletion was observed only in the recombinant alleles and thus may reflect authentic recombination, or might be caused by polymerase slippage during poly-A PCR amplification or might be due to PacBio®'s tendency for generating indels at homopolymers [69]. In conclusion, the method described here of PacBio® sequencing and analysis is appropriate for allele distribution and crossover rate only under PCR and analysis conditions that have been tested and shown to give relatively balanced allelic distribution for control plants and no crossover or indels patterns. Plants Chr3:1261146, Chr3:1228466, Chr3:1854159, Chr3:1858597 and Chr3:4639823, where selected for the DSB experiment.

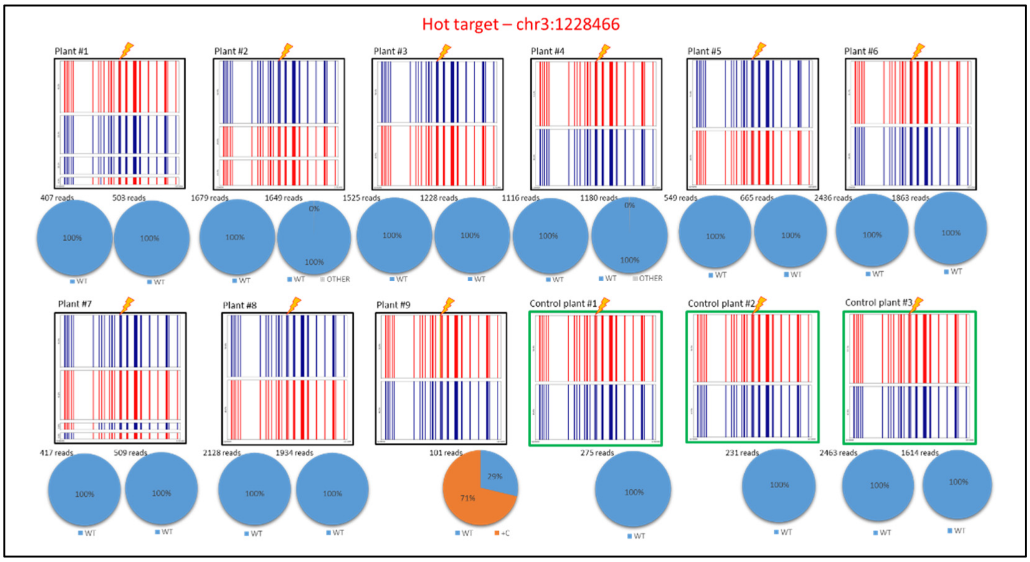


**Figure S3: Allelic distribution of the F1 plants (Ler WTx (Col (Ubi:Cas9 x U6-26:1228466 gRNA)) and control plants-** (same legend for Supplementary Figures 8-12). Every square represents the allelic distribution for one plant in~5Kb window flanking the DNA double strand break site as measured using PacBio® sequencing. F1 plants are in black squares, Control F1 Ler x Columbia tester plants in green squares. In each square, each rectangle represents one consensus sequence calculated by LAA2 analysis. Red vertical lines represent Columbia SNPs, blue vertical lines represent Landsberg SNPs, green vertical lines represent SNPs that are different from both Columbia and Landsberg SNPs, yellow line represents deletion / insertion at the break site and lightning represents the DNA DSB site. The height of the rectangle represents the frequency of this consensus sequence/allele (percentage of subreads that aligned to calculate the consensus sequence) presented always from higher to lower percentage. Pie charts under each square represent the total and distribution of Illumina-Hiseq reads for a single plant. The right pie under each square was analyzed with CRISPResso and the left analyzed with Cas-analyzer.

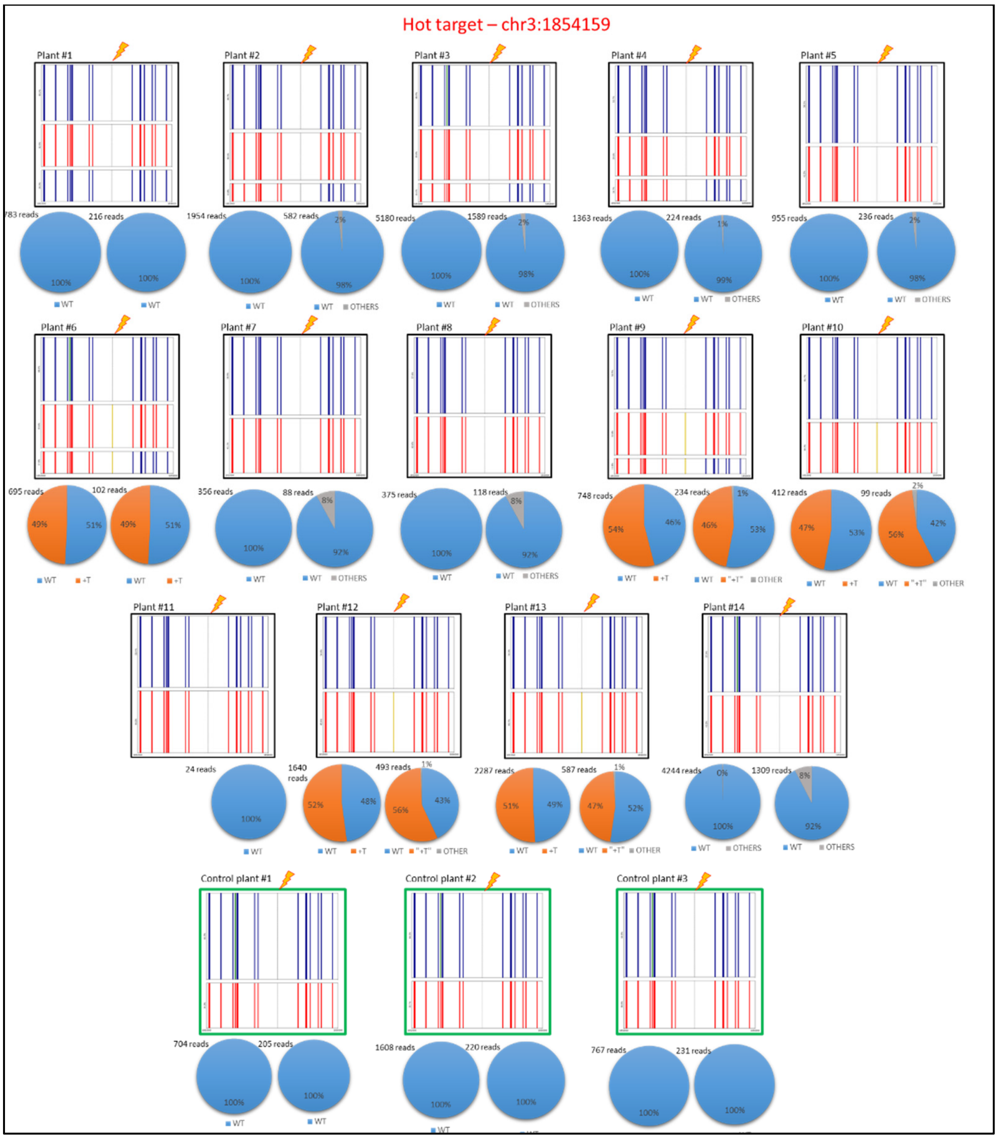


**Figure S4: Allelic distribution of the F2 plants (Ler x Ubi:Cas9 and U6-26:1854159 gRNA) and control plants.**

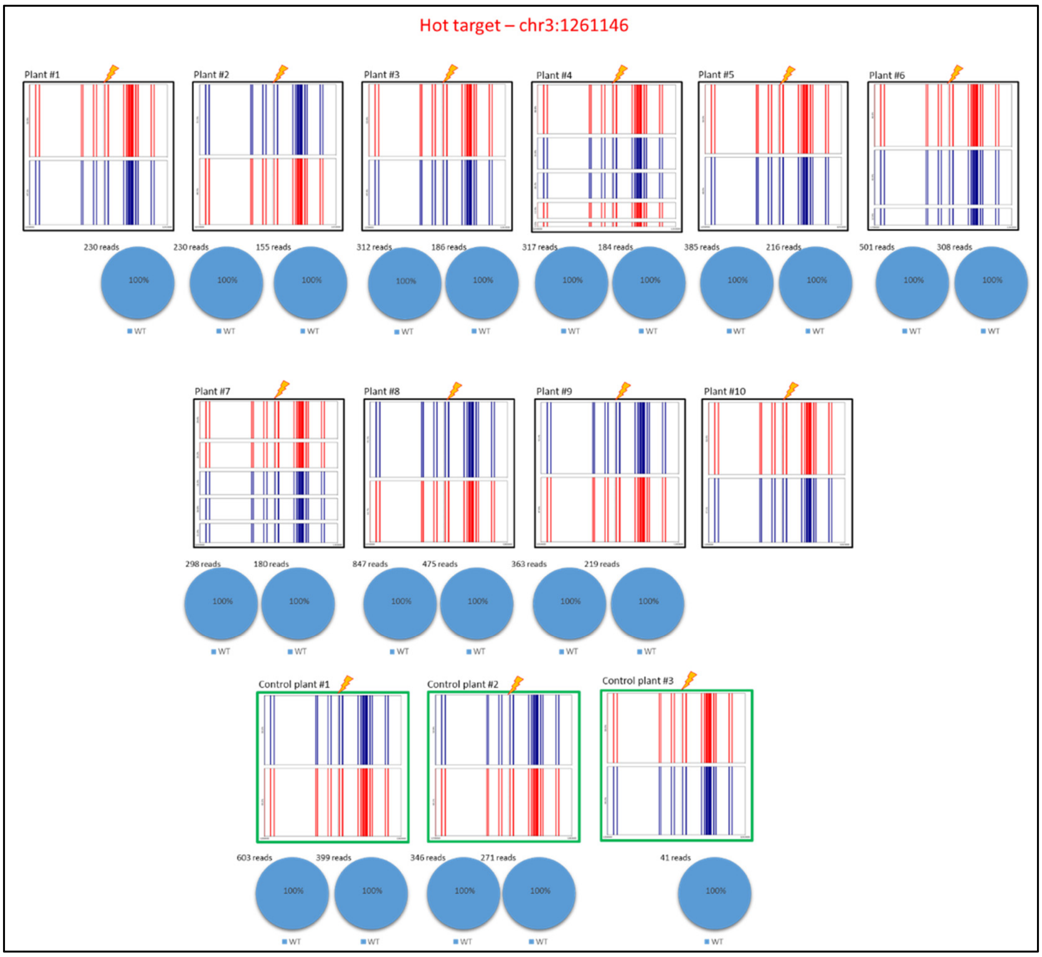


**Figure S5: Allelic distribution of the F2 plants (Ler x Ubi:Cas9 and U6-26:1261146 gRNA) and control plants.**

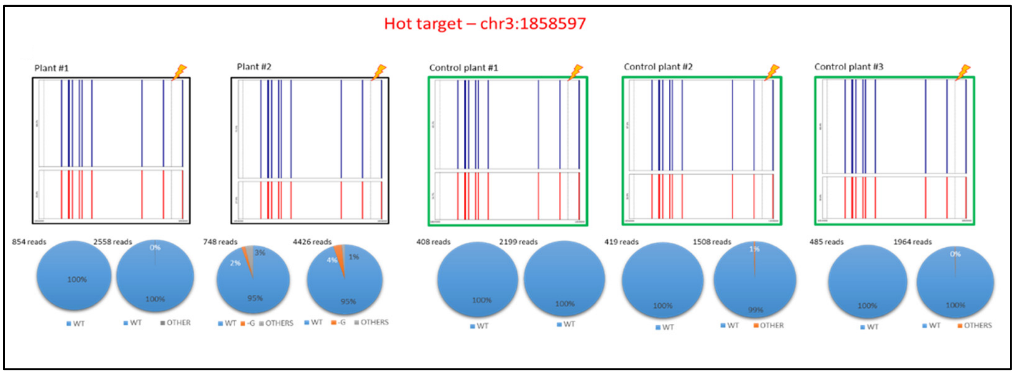


**Figure S6: Allelic distribution of the F2 plants (Ler x Ubi:Cas9 and U6-26:1858597 gRNA) and control plants.**
